# Competitive and/or Cooperative Interactions of *Listeria monocytogenes* With *Bacillus cereus* in Dual-Species Biofilm Formation

**DOI:** 10.3389/fmicb.2020.00177

**Published:** 2020-02-28

**Authors:** Vanessa Pereira Perez Alonso, Andréia Miho Morishita Harada, Dirce Yorika Kabuki

**Affiliations:** Department of Food Science, Faculty of Food Engineering, University of Campinas, Campinas, Brazil

**Keywords:** biofilm, dual-species biofilms, cooperative interactions, *Bacillus cereus*, *Listeria monocytogenes*

## Abstract

Microorganisms in dairy industries can form monospecies, dual-species, or multispecies biofilms, showing cooperative or competitive behaviors, which might contribute to the reduction of efficiency of cleaning and sanitization processes and eventually turn into a potential source of contamination. This study proposes to evaluate the behavior of *Listeria monocytogenes* in monospecies biofilms, cocultured with *Bacillus cereus*. The isolates were of dairy origin, and the selection occurred after studies of competition among species. The biofilm formations on AISI 304 stainless steel at 25°C in a stationary culture were analyzed to observe the cooperative or competitive interactions among species, as well as the effect of pre-adhered cells. Biofilm formation assays were performed in four experiments: Experiment 1: in the presence of strains of antagonistic substance producer *B. cereus* (+); Experiment 2: extract of the antagonistic substance of *B. cereus*; Experiment 3: pre-adhered cells of *B. cereus*; and Experiment 4: pre-adhered cells of *L. monocytogenes*. Subsequently, cooperative behavior was observed by scanning electron microscopy. The *L. monocytogenes* monospecies biofilm counts of greater than 5 log colony-forming units (CFU)/cm^2^ were also observed in dual-species biofilms in the presence of *B. cereus* (non-producers of antagonist substance), showing cooperative behavior between species. However, in the presence of antagonistic substance produced by *B. cereus*, the counts were lower, 1.39 and 1.70 log CFU/cm^2^ (*p* > 0.05), indicating that the antagonistic substance contributes to competitive interactions. These data are relevant for the development of new studies to control *L. monocytogenes* in the dairy industry.

## Introduction

*Listeria monocytogenes* and *Bacillus cereus* are pathogens that cause foodborne diseases and are commonly reported in the literature due the presence on environmental surfaces of dairy industries and in dairy products (Kabuki et al., 2004; Karthikeyan et al., 2015; Spanu, 2016; Saldivar et al., 2018). Chemotaxonomic markers and the analysis of 16S and 23S rRNA of *Listeria* species contributed to elucidate species position in relation to other genera of Gram-positive bacteria and its close relationship and similarity to *Bacillus* (Sallen et al., 1996; Ryser et al., 2007). Clones of *L. monocytogenes* are heterogeneous and hypervirulent and can be identified by clonal complexes as CC1, CC2, CC4, and CC6, with CC1 being strongly associated with dairy products and high clinical frequency (Maury et al., 2019). Some studies consider that the high ecological diversity of the group *B. cereus sensu lato* is related as a single evolutionary unit defined through clonal expansion and adaptation of different hosts and environments. Nevertheless, *B. cereus sensu stricto* can be characterized by diarrheal type and emetic type (Fiedoruk et al., 2017; Fayad et al., 2019).

These pathogens can produce monospecies, dual-species, or multispecies biofilms on abiotic surfaces such as stainless steel (Kim et al., 2018; Silva et al., 2018; Alonso and Kabuki, 2019). In the dairy industry, 304 stainless steel is the most common and accepted alloy in the surfaces and pipelines because of the sanitary standards and requirements (Astm International, 2019; Goff, 2019). Thus, the biofilm formations may cause biocorrosion and result in the loss of millions of dollars to the industries (Palmer et al., 2015).

Biofilms are a complex form of microbial ecosystem that allows a high level of interaction among different organisms (Saxena et al., 2019; Yuan et al., 2019). These interactions can influence both the temporal and spatial properties of biofilms. One of the consequences of interspecies interactions is the ability to exhibit greater resistance to disinfectants such as ethanol, benzalkonium chloride, sodium hypochlorite, peracetic acid, and hydrogen peroxide when compared to monospecies biofilms (Yuan et al., 2019).

Behavior among species can be cooperative, competitive, or neutral (Nadell et al., 2016; Yuan et al., 2019). In the cooperative bacterial interactions, the benefit on biofilm formations occurs by facilitating adhesion, growth, and protection against biocides. Competitive interactions may occur in the dispute for space, nutrients, and energy sources (exploitative competition), or due to the production of secondary compounds such as bacteriocins, enzymes, hydrogen peroxide, and organic acids (interference competition), favoring competitive exclusion (Elias and Banin, 2012; Knight, 2015; Saxena et al., 2019; Yuan et al., 2019).

There is a great interest in the food industry on the development of new strategies and antibiofilm agents (Møretrø et al., 2019; Ripolles-Avila et al., 2019). Bacteriophages, lysozymes, bacteriocins, antimicrobial peptides, enzymes, extracellular vesicles, lytic agents, essential oils, quorum sensing inhibitors, and surface modifications are promising alternatives (Ouertani et al., 2018; Friedlander et al., 2019; Yuan et al., 2019).

Methodologies developed based on the interaction of species to eliminate or inhibit the growth of *L. monocytogenes* in food-processing environments (Leriche et al., 1999; Zhao et al., 2004, 2006) include the use of *Listeria*-specific reagents such as *Listeria* phages and their derivatives (endolysins, bacteriocins) (Ganegama Arachchi et al., 2013; Hagens and Loessner, 2014; Gutiérrez et al., 2016; Kim et al., 2019). A recent study showed that bacteriocin pentocin MQ1 isolated from *Lactobacillus pentosus* CS2 has a biopreservative potential and presented antibacterial activity against *L. monocytogenes* and *B. cereus* (Wayah and Philip, 2018). Another example is the *Janthinobacterium* species, which inhibit the adhesion and formation of *L. monocytogenes* in mixed-species biofilms, showing that antilisterial properties can impact the microbiome in food-processing facilities (Fox et al., 2014).

Several species of *Bacillus* species are also producers of a variety of antimicrobial compounds (peptides, lipopeptides, bacteriocins, and bacteriocin-like inhibitory substances) against bacteria and fungi (Bartel et al., 2018). In a recent study, the use of *B. cereus* (AR156) in the presence of *Ralstonia solanacearum* bacteria and *Meloidogyne incognita* nematode was effective against plant diseases (Wang et al., 2019). Another study showed that *B. cereus* (RC6) was capable to secrete two enzymes that degrade casein, exhibiting antimicrobial activity to *B. cereus* ATCC 11778 (BC45) and *L. monocytogenes* DISTAM MACa1 (Ouertani et al., 2018).

The aim of this work was to evaluate the interactions of *L. monocytogenes* and *B. cereus* during the biofilm formation on stainless steel at 25°C. Understanding the competitive and/or cooperative behavior between these pathogens in dual-species biofilm is a challenge that could lead us to efficient strategies in controlling and removing biofilms (Yuan et al., 2019), contributing to a better definition of strategies in food safety (Du et al., 2018).

## Materials and Methods

### Bacterial Strains

In this study, we used 5 isolated *L. monocytogenes* and 23 *B. cereus*, all of dairy origin and which were provided by the Hygiene and Legislation Laboratory culture collection (School of Food Engineering, University of Campinas). Before each experiment, the strains were frozen at −80°C in 20% glycerol and inoculated separately into brain-heart infusion (BHI) broth (Difco, Sparks, MD, United States) at 35°C for 24 h.

### Antagonist Activity of *B. cereus* Against *L. monocytogenes*

We evaluated the production of antimicrobial substance for 23 isolates of *B. cereus* against 5 strains of *L. monocytogenes* according to the methodology used by Nascimento et al. (2009); modified methodology is reported in Supplementary Table S1. To evaluate the influence of the antimicrobial substance, the extracts of *B. cereus* were obtained. The strains were activated and inoculated separately into BHI (Difco) at 35°C for 24 h. Then, 10 mL of the cultures was incubated in a shaker (New Brunswick Scientific, Edison, NJ, United States) at 150 revolutions/min (rpm) at 30°C for 18 h and centrifuged at 10,000 × *g* for 10 min at 4°C (Model RC-5C; Sorvall Instruments, Norwalk, CT, United States). The supernatant was divided into two fractions, one without pH adjustment (approximately 5) and the other with pH between 6.5 and 7.0 with 0.1 N sodium hydroxide (NaOH) or 0.1 N HCl, and then sterilized by membrane filtration 0.22 μm (MilliporeSigma, Burlington, MA, United States). In summary, 50 μ*L*of *B. cereus* extract was inserted into 7-mm holes in BHI agar plates (0.9% wt/vol agar) (Difco) containing 1% of *L. monocytogenes* culture previously grown in BHI broth at 35°C for 18 h. Plates were incubated at 35°C for 24 h, and the inhibition halos were measured. Assays were performed on duplicates in three independent experiments.

### Biofilm Formation of *L. monocytogenes* and *B. cereus* in Static Culture

#### Surface Preparation

Stainless-steel coupons AISI 304 were used, within the no. 4 finish standard determined by the American Society for Testing and Materials with dimensions of 10 × 10 × 1 mm. The coupons were immersed in acetone for 30 min and in 1% NaOH solution for 1 h, consecutively. Next, they remained in 70% alcohol for 1 h at room temperature, rinsed in distilled water, dried at room temperature, and autoclaved at 121°C for 15 min (Parizzi et al., 2004).

#### Inocula Preparation for Biofilm

The strains used in the biofilm experiment were chosen after the evaluation of antagonistic activity (item 2.2). Cultures were as follows: *L. monocytogenes* encoded as C1-023, C1-029, E1-010, *B. cereus* producer of antagonist substance R1-070, K1-B052, M1-012, and *B. cereus* non-producer of antagonist substance E1-065, M1-016, M1-026. For the monospecies biofilm assays, three strains of each species [*L. monocytogenes*, *B. cereus* producer of antagonist substance (*B. cereus* +), and *B. cereus* non-producer of antagonist substance (*B. cereus* −)] were activated separately in BHI broth (Difco) at 35°C for 18 to 24 h. Then, 2 mL of each species was mixed, resulting in a pool of 6 mL and serially diluted in peptone water (0.1% wt/vol; Difco) until reaching the concentration of approximately 4 log colony-forming units (CFU)/mL. For dual-species biofilm assays, 2 mL of each species pool previously prepared was mixed and diluted until reaching the concentration required according to the purpose of the experiment.

#### Dual-Species Biofilm Setup

**FIGURE 1:**
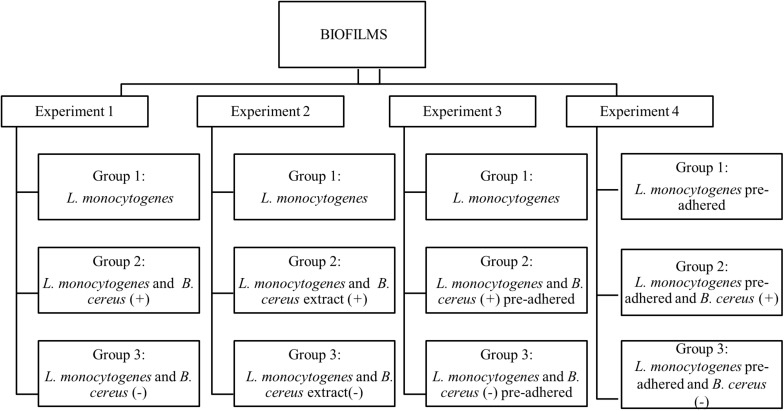
Flowchart of dual-species biofilm formation in stationary culture on stainless steel surface in BHI kept at 25°C. *B. cereus* producer of antagonist substance (+) and *B. cereus* non-producer antagonist substances (–).

The observations of interactions (cooperative or competitive) were performed in four experiments on stainless-steel coupons at 25°C for 7 days. All experiments (1, 2, 3, and 4) were composed of three groups (Figure 1). Two independent coupons were used for each day, and all experiments were conducted in triplicates. To form the biofilm, the inoculum was added in 60 mL of BHI broth to obtain a final concentration of 3 log CFU/mL, and then the coupons were aseptically immersed.

Experiment 1. Herein, the biofilms were formed from vegetative cells, and inoculum was produced according to each group. Group 1: used as a control, formed only by *L. monocytogenes* biofilm; Group 2: influence of vegetative cells of *B. cereus* (+) in the biofilm dual-species with *L. monocytogenes*; Group 3: biofilm dual-species in the presence *B. cereus* (−).

Experiment 2. The interference of the antagonist substance produced by *B. cereus* on *L. monocytogenes* monospecies biofilm formation was evaluated, adding approximately 20 mL of *B. cereus* extract in 40 mL BHI broth. Group 1: *L. monocytogenes* biofilm (control group); Group 2: herein, 20 mL of *B. cereus* (+) extract was used on *L. monocytogenes* biofilm; Group 3: 20 mL of *B. cereus* (−) extract was used on *L. monocytogenes* biofilm.

Experiment 3. The behavior of pre-adhered *B. cereus* cells on stainless steel on *L. monocytogenes* biofilm was assessed. For the adhesion of vegetative cells, the coupons were immersed in BHI broth containing 5 log CFU/mL of inoculum at 25°C for 6 h until approximately 3 log CFU/cm^2^, and then rinsed in 10 mL peptone water (0.1% wt/vol). Group 1: *L. monocytogenes* biofilm (control group); Group 2: pre-adhered *B. cereus* (+) on coupons immersed in *L. monocytogenes* inoculum; Group 3: pre-adhered *B. cereus* (−) immersed in *L. monocytogenes* inoculum.

Experiment 4. Pre-adhered *L. monocytogenes* was carried out by the same procedure as Experiment 3. Group 1: *L. monocytogenes* biofilm (control group); Group 2: coupons with pre-adhered *L. monocytogenes* immersed in *B. cereus* (+) inoculum; Group 3: pre-adhered *L. monocytogenes* on coupons immersed in *B. cereus* (−) inoculum.

Substrate exchange (BHI) occurred on the second and fourth days for all experiments, but for Experiment 2, the antagonist substance was not added again.

#### Count of *L. monocytogenes* and *B. cereus* on Stainless-Steel Coupons

In the days of incubation (1, 2, 4, and 7), two coupons of each group were removed from the culture medium and evaluated. Coupons were immersed separately in 10 mL peptone solution (0.1% wt/vol) for 1 min to remove the planktonic cells. Then, coupons were transferred to 5 mL saline solution (0.85% NaCl wt/vol) and vortexed (2800 rpm) for 2 min to remove the sessile cells (Andrade et al., 1998). The suspensions were diluted and spread plated on BHI agar and MYP (mannitol–egg yolk–polymyxin) for the enumeration of *L. monocytogenes* and *B. cereus*, respectively. The colonies of *L. monocytogenes* on BHI agar were differentiated by size and morphological characteristics. The plates were incubated at 35°C for 48 h. Then, the colonies were counted, and the results expressed as log CFU/cm^2^.

### Statistical Analysis

Variables related to biofilm formation and the interactions of species were evaluated by analysis of variance, and differences were compared using the Tukey test at 5% significance level, using the software Statistic version 8.0 (StatSoft, Tulsa, OK, United States).

### Scanning Electron Microscopy

Coupons were analyzed by scanning electron microscopy (SEM) according to the protocol proposed by Lou et al. (2013).

The coupons were immersed in peptone water (0.1% wt/vol) for removal of non-adhered cells. To fix the biofilm, 2 mL of 0.1 M phosphate-buffered solution (Sigma-Aldrich, St. Louis, MO, United States), supplemented with 2% (wt/vol) glutaraldehyde (Sigma-Aldrich), was used. Samples were rinsed in 0.1 M phosphate-buffered solution, followed by dehydration with ethanol P.A. (Merck SA Chemical Industries, Rio de Janeiro, Brazil) at increasing concentrations (30, 50, 70, 80, and 95%) and three times in ethanol 100% for 10 min. Then, these were transferred to the critical point dryer (model CPD 030; Balzers, Liechtenstein) using CO^2^ for complete removal of ethanol. After drying, the coupons were coated with a metal layer in the apparatus “Sputter Coater” (model SCD 050; Balzers, Liechtenstein) and analyzed by SEM (model JSM 5800LV; JEOL, Tokyo, Japan; and Phenom-FEI Company, Hillsboro, OR, United States) (Lou et al., 2013).

The images obtained by SEM were analyzed by ImageJ version 1.52N (National Institutes of Health, Bethesda, MD, United States).

## Results and Discussion

### Evaluation of Antagonistic Activity of *B. cereus* Against *L. monocytogenes*

Three of 23 *B. cereus* cultures showed activity >2.0-cm diameter of inhibition zone, thus considered strong producers of antagonistic substances: R1-070, K1-B052, and M1-012. This result evidences a low occurrence (13%) of *B. cereus* capable of producing an antimicrobial substance in dairy products. The inhibition halos varied from 2 to 12 mm (Table 1). Culture of *B. cereus* M1-012, isolated from pâté cheese, showed the highest inhibitory effect (12 mm).

**TABLE 1:** Diameters average of inhibition halos of *Listeria monocytogenes* strains by *Bacillus cereus*.

***B. cereus***	***L. monocytogenes***					
**Identification**	**C1-023 (mm)**	**SD**	**C1-029 (mm)**	**SD**	**E1-010 (mm)**	**SD**
R1-070	4	±0.23	4	±0.23	2	±0.12
R1-070*	8	±0.20	10	±0.31	6	±0.20
K1-B052	4	±0.31	4	±0.23	2	±0.12
K1-B052*	8	±0.31	10	±0.20	6	±0.20
M1-012	10	±0.23	10	±0.12	8	±0.31
M1-012*	12	±0.35	12	±0.42	10	±0.20

*With pH adjustment (6.5–7.0). SD: ± standard deviation. Origin of the strains: *B. cereus* R1-070 (Minas Frescal Cheese), K1-B052 (Minas Frescal Cheese), and M1-012 (Ricotta Pâté) and *L. monocytogenes* C1-023 (Minas Frescal Cheese), C1-029 (Coalho Cheese), and E1-010 (Ricotta).

Other studies demonstrated the effectiveness of *Bacillus* species against fungal pathogens in corn (*Fusarium verticillioides*) (Douriet-Gámez et al., 2018), bacteria (*R. solanacearum*), and nematodes (*M. incognita*) disease in tomato (Wang et al., 2019). Peptides and bacteriocins produced by this species may also inhibit *L. monocytogenes*, *Bacillus subtilis*, *Escherichia coli*, and *Salmonella enteritidis*, *Salmonella typhi*, and *Staphylococcus aureus* (Bizani et al., 2005; Yusra and Novelina, 2014). These observations are important on the development of solutions to eliminate biofilms in the food industry (Liu et al., 2016; Ouertani et al., 2018).

### Monospecies Biofilm Formation of *L. monocytogenes*

**FIGURE 2:**
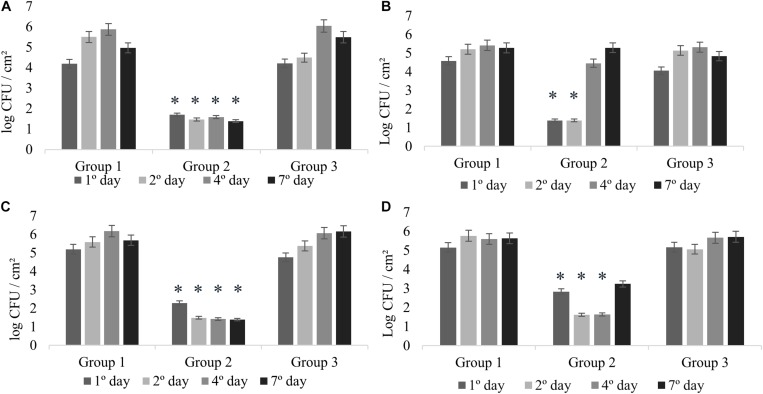
Counts of *L. monocytogenes* at 25°C expressed in log CFU/cm^2^. Experiment 1 **(A)** in presence of *B. cereus* producer and non-producer of antagonistic substance; Experiment 2 **(B)** in presence of antimicrobial extract; Experiment 3 **(C)** in presence of *B. cereus* pre-adhered; and Experiment 4 **(D)** in presence of *L. monocytogenes* pre-adhered. ^∗^*p* < 0.05.

All the experiments of monospecies biofilms (Group 1) were used as control for comparison with the other assays reported in this study (Figure 1). *L. monocytogenes* formed monospecies biofilm in Experiments 1 and 2 on the second sampling day (Figures 2A,B) and in Experiments 3 and 4 on the first day. In Experiment 4, there was a modification in the control group: the biofilm was made after pre-adherence of *L. monocytogenes* in order to check for possible interference in the biofilm formation. Despite that, when compared to the other experiments, there were no differences (*p* > 0.05).

In agreement with previous studies, *L. monocytogenes* isolated from milk and dairy products are able to form monospecies biofilm (Alonso and Kabuki, 2019; Skowron et al., 2019). Agglomerates with counts greater than 5 log CFU/cm^2^ were considered biofilms in a maturity stage because this threshold demonstrates that bacteria reached the three-dimensional complexity in their structure surrounded by extracellular polymeric substances (EPS) (Ronner and Wong, 1993).

### Experiment 1: Dual-Species Biofilm Formation of *L. monocytogenes* and *B. cereus*

The interactions in dual-species biofilms (Experiment 1) were analyzed in the presence of *B. cereus* producer of antagonistic substance (+) (Group 2) and *B. cereus* non-producer of antagonistic substance (−) (Group 3). In Group 2, *L. monocytogenes* showed adherence on stainless steel with counts between 1.70 and 1.39 log CFU/cm^2^ (Figure 2A), which is not considered biofilm formation (Ronner and Wong, 1993). Therefore, the interaction for this group can be considered as competitive, in which the antagonistic substance inhibited the biofilm formation of *L. monocytogenes*, showing antagonistic interactions among both species. On the other hand, the results of Group 3 revealed cooperative interactions with a statistically significant difference (*p* < 0.05) presenting counts between 6.04 and 5.49 log CFU/cm^2^ (Figure 2A), as evidenced by the control group (between 5.50 and 5.87 log CFU/cm^2^) (Figure 2A). These results demonstrate that the bacterial diversity present in foods may behave differently, thus enabling competitive or cooperative interactions, further corroborating with other studies (Fox et al., 2014).

Interactions among species have an important purpose in the formation, structure, and function of bacterial communities (Liu et al., 2017). According to the literature, other species are also capable of inhibiting the growth of *Listeria*, for example, *Pseudomonas graminis* CPA-7, *Lactobacillus plantarum* Tennozu-SU2, and *Lactococcus lactis* subsp. *lactis* BF1 (Collazo et al., 2019; Haraguchi et al., 2019). Some studies reported the opposite results in dual-species biofilms, such as *Flavobacterium* species and *Pseudomonas*, known for increasing attachment of *L. monocytogenes* (Sasahara and Zottola, 1993; Bremer et al., 2001). This behavior is due to the protection of the species among themselves or to the induction of specific tolerance phenotypes as a response to competitors (Parijs and Steenackers, 2018). Interactions in biofilms may also be associated with the acquisition of conjugation genes due to lateral gene transfer (Saraoui et al., 2016). Usually, strains that improve colonization and biofilm formation contribute to the persistence of *L. monocytogenes* in food industry plants and are a potential risk to food safety (Giaouris et al., 2015).

### Experiment 2: Effect of the *B. cereus* Extract in Biofilm Formation of *L. monocytogenes*

In this experiment, the possible effects of antagonist substance produced by *B. cereus* (Group 2) on *L. monocytogenes* biofilm formation were verified, comparing with the non-antagonistic substance producer strains (Group 3). The counts of *L. monocytogenes* for Group 2 on the first and second days were less than 1.39 log CFU/cm^2^ (Figure 2B), evidencing the antibiofilm activity of the antagonistic substance, whereas in the control group, biofilm formation was 5.21 log CFU/cm^2^ (Figure 2B). This result is in agreement with that obtained in the antagonistic competition between individual species (see section “Experiment 1: Dual-Species Biofilm Formation of *L. monocytogenes* and *B. cereus*”).

There was a difference in *L. monocytogenes* counting (*p* < 0.05) of approximately 3 and 4 logarithmic cycles between Group 2 and Group 3 results (Figures 2B,C). After the second day, the culture medium (BHI) was changed; however, the substance was not replaced, so that we could observe how *L. monocytogenes* would behave after interference. Counts reached 4.46 and 5.29 log CFU/cm^2^ at fourth and seventh days, respectively.

The behavior was similar for Experiments 1 and 2 (Figures 2A,B), indicating the antibiofilm activity of *B. cereus* against *L. monocytogenes*. The inhibition observed in Experiment 1 might be related to interference competition rather than exploitative competition, due to the production of secondary compounds or antimicrobial agents that facilitate competitor dispersal (Ouertani et al., 2018; Yuan et al., 2019). Antagonistic response depends on several factors, such as strain-specific response, substrates, and the interaction between species (Bartel et al., 2018). In general, the antagonistic assays can offer an alternative to the indiscriminate use of chemicals, and therefore it has attracted increasing interest from researchers (Liu et al., 2017; Bartel et al., 2018).

### Experiments 3 and 4: Effect of Pre-adhered Cells in Dual-Species Biofilm Formation

Pre-adhered cells of *B. cereus* and *L. monocytogenes* on coupons were used to evaluate the possible change of behavior in the species in relation to the inhibitory effect. Eventual increase or reduction was compared with Experiments 3 and 4 (Figures 2C,D) and also with Experiment 1, when species were inserted simultaneously.

In Experiments 3 and 4, competitive interactions were observed in Group 2, confirming the result found in Experiment 1. In these groups, the counts were between less than 1.39 to 2.29 log CFU/cm^2^ (Experiment 3) and 1.62 to 3.24 log CFU/cm^2^ (Experiment 4). Although *L. monocytogenes* cells adhered, the count did not reach the minimum value to be considered biofilm (5 log CFU/cm^2^) (Ronner and Wong, 1993). This behavior confirms that the presence of antagonistic substance reduces competition and affects the prevalence of some species (Parijs and Steenackers, 2018).

In Group 3, in the presence of pre-adhered *B. cereus* (−) (Experiment 3; Figure 2C) and pre-adhered *L. monocytogenes* (Experiment 4; Figure 2D), we observed the cooperative interaction. The counts were similar to those of Group 1, varying between 4.76 and 6.16 log CFU/cm^2^ (Experiment 3) and between 5.16 and 5.71 log CFU/cm^2^ (Experiment 4).

In dual-species biofilm, the difficulty of *L. monocytogenes* to form biofilms can be affected by physicochemical characteristics of contact surfaces and the hydrophilic properties of strains (Giovannacci et al., 2000), showing greater difficulty to interact with the stainless-steel surface, also considered hydrophilic (Bos et al., 2000). On the other hand, the cells of *B. cereus* exhibit hydrophobic properties, facilitating the interaction with stainless steel (hydrophilic) (Bos et al., 2000). Therefore, hydrophobicity is also correlated with aggregation of cells (Park-ji et al., 2019).

The inhibition of some multispecies biofilm could be due to the quorum sensing system, production of antibacterial substances (Jahid et al., 2019), and the presence of organic compounds, such as dairy substrates, which may lead to dispersion by seeding (Alonso and Kabuki, 2019; Jahid et al., 2019).

These results contribute to the knowledge on the behavior of bacterial strain interactions in dual-species biofilm, showing that interactions can contribute to competition and cooperation, increasing antimicrobial tolerance (Parijs and Steenackers, 2018).

### Structure of Dual-Species Biofilms in SEM

**FIGURE 3:**
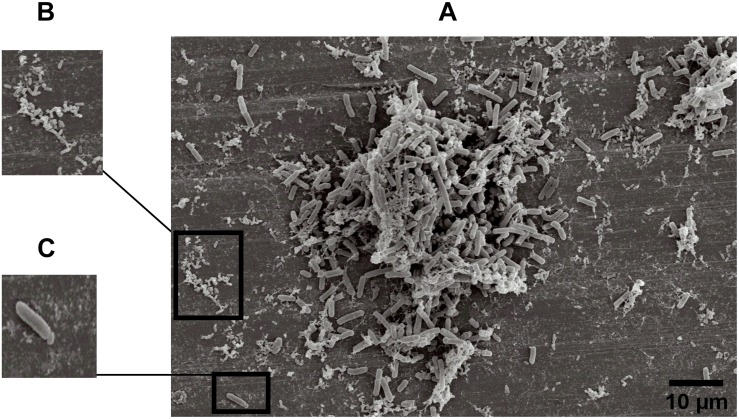
Image obtained by SEM (JEOL) of microbial cooperative interaction of *L. monocytogenes* and *B. cereus* (+). Experiment 1, Group 3, on stainless steel **(A)**; *L. monocytogenes* **(B)**; and *B. cereus* **(C)**.

**FIGURE 4:**
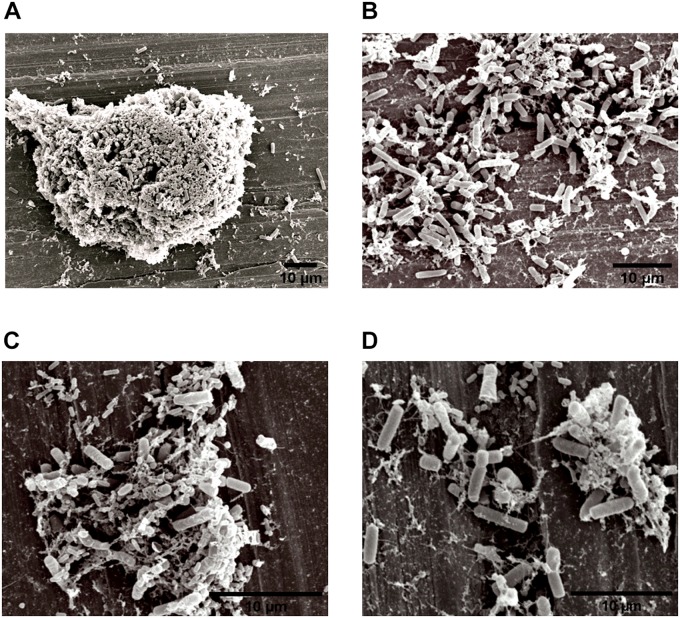
Image obtained by SEM (JEOL) of the interaction between *L. monocytogenes* and *B. cereus* in the biofilm on stainless steel (Experiment 1, Group 3). Agglomerates of dual-species biofilms **(A)**; Biofilm cells **(B,C)**; and low EPS production **(D)**.

The specific spatial arrangement of the dual-species biofilm was observed by SEM for Experiment 1–Group 3 on the fourth day at 25°C. The cooperative interaction in dual-species biofilms between *L. monocytogenes* (Figure 3B) and *B. cereus* (Figure 3C) was easily visualized in microscopy (Figures 3, 4) due to the formation of multilayer cells (Kalmokoff et al., 2001). The size of cells and bacterial agglomerates on stainless steel was analyzed by ImageJ software.

In the cell size analysis, *n* = 3 were measured. We observed that *B. cereus* cells presented lengths ranging from 2.23 to 6.92 ± 0.75 μm and diameters from 1.00 to 1.33 ± 0.07 μm, whereas *L. monocytogenes* were measured with lengths from 1.08 to 1.55 ± 0.07 and 0.46 to 0.70 ± 0.05 μm in diameter. According to the SEM measurements, the area of the formed aggregates showed values between 1616.7 and 33,199.7 μm^2^ (Supplementary Figure S1). Although elongated cells have been reported in biofilms of other species (Janissen et al., 2015), in this experiment both species (*B. cereus* and *L. monocytogenes*) had the common planktonic cell size (Logan and De Vos, 2015; Ludwig et al., 2015).

Three types of bacterial organization are known in biofilms: microcolonies, forming separate small niches side by side (Nielsen et al., 2000); layered structures, where each species is located in a different layer (Habimana et al., 2010); and niches with mixed species (Liu et al., 2018). In SEM experiment, the formation of niches with mixed species was observed, as shown in Figures 3, 4 in which microorganisms coexist in dual-species biofilms incorporated with EPS matrix (Liu et al., 2018; Yuan et al., 2019).

The organization of the biofilm structure is not random and might favor the survival of present species, which reveals the relevance of the interactive nature between them. These organizations allow both competitive and cooperative interactions (An et al., 2006; Habimana et al., 2010) influencing the survival rate of species in unfavorable environments (Nadell et al., 2016).

In dual-species biofilms, a small amount of EPS was observed by the microscope (Figures 3, 4). This may possibly be justified by the presence of *L. monocytogenes*, because it is often reported as a few EPS producers (Doijad et al., 2015) compared to *B. cereus* (Park-ji et al., 2019). However, this small amount is sufficient for both species to survive in the biofilm (Park-ji et al., 2019).

The EPS provides protection and trap nutrients for microorganisms in the biofilm, contributing to the formation of the cluster (Figure 4A). However, the adhesion of bacteria on surfaces is not related to the production of EPS, because adhesion and consequent biofilm formation can occur even in the absence of the polymeric substance (Hoodt and Zottola, 1997). Despite lack of information on the molecular mechanisms, such as genes and EPS characteristics involved in biofilm formation (Park-ji et al., 2019), there are reports in the literature that EPS production depends on stress and environmental conditions (Giaouris et al., 2015).

The adhesion of *B. cereus* to surfaces also contributes to the formation of a conditioning layer, facilitating subsequent adhesion of other bacteria (Marchand et al., 2012), and the capability to form biofilms under static conditions (Wijman et al., 2007), because of their hydrophobic properties (Park-ji et al., 2019).

Herald and Zottola (1988) and Hoodt and Zottola (1997) found that *Listeria* species and *Yersinia* species showed an increased adhesion capacity during high metabolic activity. Thus, it is also possible that in our experiments the microorganisms were in this phase at the fourth day of incubation, facilitating the visualization of the species on the stainless steel in Group 3 (Figures 3, 4).

## Conclusion

The *L. monocytogenes* strains isolated from dairy products showed the capacity of cooperative and competitive interactions in dual-species biofilm with *B. cereus*. Cooperative interaction in mixed species showed that bacterial spatial organization coexists in the same matrix incorporated with EPS, corroborated by SEM measurements. Moreover, competitive interaction was dependent on the ability of *B. cereus* to produce metabolites that showed antibiofilm action against *L. monocytogenes.* Specific and in-depth studies on the biochemical characterization of antimicrobial substances produced by *B. cereus* may be the subject of further research.

## Data Availability Statement

The raw data supporting the conclusions of this manuscript will be made available by the authors, without undue reservation, to any qualified researcher.

## Author Contributions

VA and AH performed the experimental stages, the statistical analyzes, and wrote the manuscript. VA wrote, adapted to the guidelines, reviewed, and finalized the manuscript. DK provided and designed the experiments, supervised all the steps, and reviewed the manuscript.

## Conflict of Interest

The authors declare that the research was conducted in the absence of any commercial or financial relationships that could be construed as a potential conflict of interest.
