# Supplementary material for: Competitive and/or Cooperative Interactions of Listeria monocytogenes With Bacillus cereus in Dual-Species Biofilm Formation
Source: Front Microbiol. 2020 Feb 28;11:177. doi: 10.3389/fmicb.2020.00177 (PMC7058548; doi:10.3389/fmicb.2020.00177)
Supplement: Supplementary file 1 [file Data_Sheet_1.pdf]

Supplementary Material

Supplementary Table S1. Diameters average of inhibition halos of *L. monocytogenes* strains by *B. cereus*

Bacillus cereus	*Listeria monocytogenes*				
Identification	C1-023	C1-029	E1-008	E1-010	M1-003
E1-051	0 mm (±0.00) [a]	0 mm (±0.00)	0 mm (±0.00)	0 mm (±0.00)	0 mm (±0.00)
E1-051*	0 mm (±0.00)	0 mm (±0.00)	0 mm (±0.00)	0 mm (±0.00)	0 mm (±0.00)
E1-065	0 mm (±0.00)	0 mm (±0.00)	0 mm (±0.00)	0 mm (±0.00)	0 mm (±0.00)
E1-065*	0 mm (±0.00)	0 mm (±0.00)	0 mm (±0.00)	0 mm (±0.00)	0 mm (±0.00)
E1-075	0 mm (±0.00)	0 mm (±0.00)	0 mm (±0.00)	0 mm (±0.00)	0 mm (±0.00)
E1-075*	0 mm (±0.00)	0 mm (±0.00)	0 mm (±0.00)	0 mm (±0.00)	0 mm (±0.00)
K1-B021	0 mm (±0.00)	0 mm (±0.00)	0 mm (±0.00)	0 mm (±0.00)	0 mm (±0.00)
K1-B021*	0 mm (±0.00)	0 mm (±0.00)	0 mm (±0.00)	0 mm (±0.00)	0 mm (±0.00)
K1-B025	0 mm (±0.00)	0 mm (±0.00)	0 mm (±0.00)	0 mm (±0.00)	0 mm (±0.00)
K1-B025*	0 mm (±0.00)	0 mm (±0.00)	0 mm (±0.00)	0 mm (±0.00)	0 mm (±0.00)
K1-B050	0 mm (±0.00)	0 mm (±0.00)	0 mm (±0.00)	0 mm (±0.00)	0 mm (±0.00)
K1-B050*	0 mm (±0.00)	0 mm (±0.00)	0 mm (±0.00)	0 mm (±0.00)	0 mm (±0.00)
K1-B052	4 mm (±0.31)	4 mm (±0.23)	1 mm (±0.23)	2 mm (±0,12)	1 mm (±0.23)
K1-B052*	8 mm (±0.20)	10 mm (±0.20)	4 mm (±0.42)	6 mm (±0.20)	6 mm (±0.53)
K1-B056	0 mm (±0.00)	0 mm (±0.00)	0 mm (±0.00)	0 mm (±0.00)	0 mm (±0.00)
K1-B056*	0 mm (±0.00)	0 mm (±0.00)	0 mm (±0.00)	0 mm (±0.00)	0 mm (±0.00)
K1-B059	0 mm (±0.00)	0 mm (±0.00)	0 mm (±0.00)	0 mm (±0.00)	0 mm (±0.00)
K1-B059*	0 mm (±0.00)	0 mm (±0.00)	0 mm (±0.00)	0 mm (±0.00)	0 mm (±0.00)
K1-B061	0 mm (±0.00)	0 mm (±0.00)	0 mm (±0.00)	0 mm (±0.00)	0 mm (±0.00)
K1-B061*	0 mm (±0.00)	0 mm (±0.00)	0 mm (±0.00)	0 mm (±0.00)	0 mm (±0.00)
M1-012	10 mm (±0.23)	10 mm (±0.12)	4 mm (±0.35)	8 mm (±0.31)	5 mm (±0.46)
M1-012*	12 mm (±0.35)	12 mm (±0.42)	3 mm (±0.42)	10 mm (±0.20)	7 mm (±0.64)

M1-013	0 mm (±0.00)	0 mm (±0.00)	0 mm (±0.00)	0 mm (±0.00)	0 mm (±0.00)
M1-013*	0 mm (±0.00)	0 mm (±0.00)	0 mm (±0.00)	0 mm (±0.00)	0 mm (±0.00)
M1-016	0 mm (±0.00)	0 mm (±0.00)	0 mm (±0.00)	0 mm (±0.00)	0 mm (±0.00)
M1-016*	0 mm (±0.00)	0 mm (±0.00)	0 mm (±0.00)	0 mm (±0.00)	0 mm (±0.00)
M1-017	0 mm (±0,.0)	0 mm (±0,.0)	0 mm (±0,.0)	0 mm (±0,.0)	0 mm (±0,.0)
M1-017*	0 mm (±0.00)	0 mm (±0.00)	0 mm (±0.00)	0 mm (±0.00)	0 mm (±0.00)
M1-021	0 mm (±0.00)	0 mm (±0.00)	0 mm (±0.00)	0 mm (±0.00)	0 mm (±0.00)
M1-021*	0 mm (±0.00)	0 mm (±0.00)	0 mm (±0.00)	0 mm (±0.00)	0 mm (±0.00)
M1-024	0 mm (±0.00)	0 mm (±0.00)	0 mm (±0.00)	0 mm (±0.00)	0 mm (±0.00)
M1-024*	0 mm (±0.00)	0 mm (±0.00)	0 mm (±0.00)	0 mm (±0.00)	0 mm (±0.00)
M1-026	0 mm (±0.00)	0 mm (±0.00)	0 mm (±0.00)	0 mm (±0.00)	0 mm (±0.00)
M1-026*	0 mm (±0.00)	0 mm (±0.00)	0 mm (±0.00)	0 mm (±0.00)	0 mm (±0.00)
M1-028	0 mm (±0.00)	0 mm (±0.00)	0 mm (±0.00)	0 mm (±0.00)	0 mm (±0.00)
M1-028*	0 mm (±0.00)	0 mm (±0.00)	0 mm (±0.00)	0 mm (±0.00)	0 mm (±0.00)
R1-070	4 mm (±0.23)	4 mm (±0.23)	0 mm (±0.00)	2 mm (±0.12)	0 mm (±0.00)
R1-070*	8 mm (±0.20)	10 mm (±0.31)	1 mm (±0.23)	6 mm (±0.00)	2 mm (±0.46)
R1-097	0 mm (±0.00)	0 mm (±0.00)	0 mm (±0.00)	0 mm (±0.00)	0 mm (±0.00)
R1-097*	3 mm (±0.31)	2 mm (±0.35)	2 mm (±0.35)	6 mm (±0.12)	0 mm (±0.00)
R1-130	0 mm (±0.00)	0 mm (±0.00)	0 mm (±0.00)	0 mm (±0.00)	0 mm (±0.00)
R1-130*	0 mm (±0.00)	0 mm (±0.00)	0 mm (±0.00)	0 mm (±0.00)	0 mm (±0.00)
156	0 mm (±0.00)	0 mm (±0.00)	0 mm (±0.00)	0 mm (±0.00)	0 mm (±0.00)
156*	0 mm (±0.00)	0 mm (±0.00)	0 mm (±0.00)	0 mm (±0.00)	0 mm (±0.00)
181	0 mm (±0.00)	0 mm (±0.00)	0 mm (±0.00)	0 mm (±0.00)	0 mm (±0.00)
181*	0 mm (±0.00)	0 mm (±0.00)	0 mm (±0.00)	0 mm (±0.00)	0 mm (±0.00)

*= with pH adjustment (6,5 – 7,0); [a] (SD): ± Standard Deviation

Origin of the strains: *B. cereus:* E1-051 (Ricotta), E1-065 (Ricotta), E1-075 (Ricotta), K1-B021 (Minas Frescal Cheese), K1-B025 (Minas Frescal Cheese), K1-B050 (Minas Frescal Cheese), K1-B052 (Minas Frescal Cheese), K1-B056 (Ricotta), K1-B059 (Ricotta), K1-B061 (Minas Frescal Cheese), M1-012 (Ricotta Pâté), M1-013 (Pasteurized Dairy Cream), M1-016 (Pasteurized Dairy Cream), M1-017 (Pasteurized Dairy Cream), M1-021 (Ice Cream), M1-024 (Pasteurized Dairy Cream), M1-026 (Pasteurized Heavy Cream), M1-028 (Cheese Pâté), R1-070 (Minas Frescal Cheese), R1-097 (Minas Frescal Cheese), R1-130 (Minas Frescal Cheese), 156 (Ricotta), 181 (Ricotta). *L. monocytogenes* C1-023 (Minas Frescal Cheese), C1-029 (Coalho Cheese), E1-008 (Ricotta) E1-010 (Ricotta) and M1-003 (Pasteurized Dairy Cream). SD: ± Standard Deviation

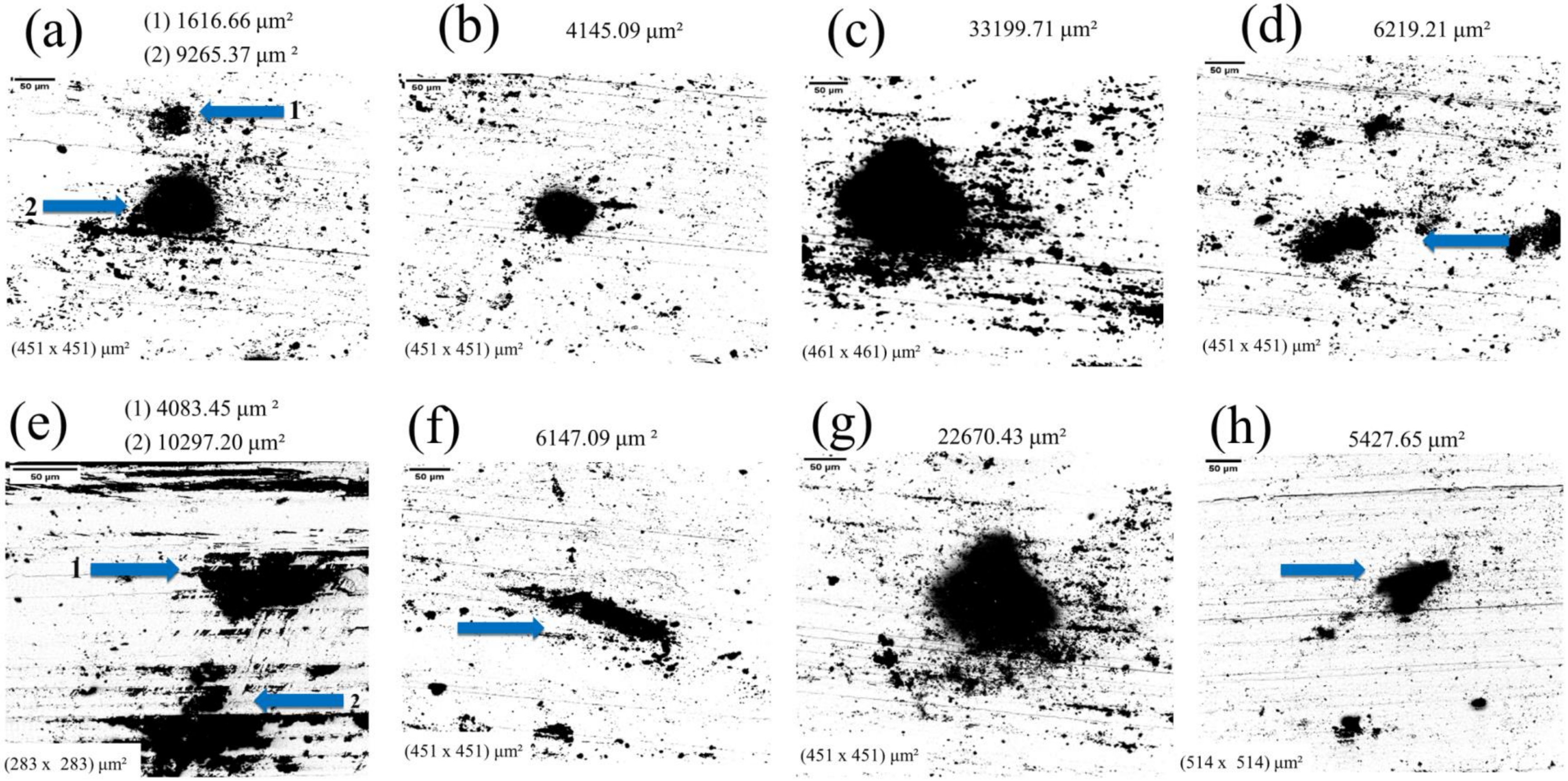

Supplementary Figure S1. Measured area of dual-species biofilm on stainless steel obtained by SEM (Phenom). Blue arrows indicate the agglomerates analyzed by ImageJ
